# An Interesting Case of Recurrent Postprandial Cardiogenic Syncope Caused by Type III Hiatal Hernia

**DOI:** 10.7759/cureus.47791

**Published:** 2023-10-27

**Authors:** Ammad J Chaudhary, Muhammad H Qureshi, Husam El Sharu, Jonathan Prostak

**Affiliations:** 1 Internal Medicine, Henry Ford Health System, Detroit, USA; 2 Internal Medicine, Mayo Hospital, Lahore, PAK; 3 Internal Medicine, East Carolina University, Greenville, USA

**Keywords:** diagnosis, atrial compression, gastroesophageal reflux disease (gerd), hiatal hernia, syncope

## Abstract

Patients with syncope often present a diagnostic challenge due to the diverse causes of this condition. While a careful assessment can identify the underlying cause in many cases, syncope can arise from a variety of sources, including structural anomalies. Among these, hiatal hernia (HH) is a relatively common yet unusual condition associated with syncope. HH involves the protrusion of abdominal organs into the mediastinum through the diaphragmatic esophageal hiatus, with types III and IV being capable of causing cardiac problems. We report a case of a 92-year-old patient with a known HH history who experienced recurrent syncope episodes triggered by heavy meals. Extensive evaluation ruled out cardiac and neurological causes. Imaging revealed a large HH compressing the left atrium. Despite being an infrequent occurrence, such cases highlight the potential for atrial compression-induced syncope, which can be effectively managed with proton pump inhibitors and lifestyle modifications, as demonstrated by our patient's positive outcome.

## Introduction

Patients presenting with syncope can occasionally pose a diagnostic challenge due to the numerous causes of the complaint. A careful history and physical examination can narrow down the cause of syncope in approximately half of the patients [1]. Syncope is often linked to cardiovascular issues, particularly arrhythmias. In the elderly, 10% to 30% of syncopal events can be traced back to a cardiac cause. Ventricular tachycardia alone accounts for 11% of these cases. Additionally, structural cardiac diseases can also cause syncope but are more prevalent among individuals with comorbidities like diabetes, hypertension, and hyperlipidemia, as well as among smokers [2]. On rare occasions, extracardiac structural anomalies can result in symptoms of syncope as well. Among them, a rather common but unusual structural anomaly associated with syncope is a hiatal hernia [3].

Hiatal hernia (HH) is the protrusion of abdominal organs into the mediastinum through the diaphragmatic esophageal hiatus. HH can be of four different types. The majority of hiatal hernias, specifically type 1 or sliding hiatal hernias, constitute more than 95% of all cases. Types II through IV are classified as paraesophageal hernias, with type II involving the normal position of the gastroesophageal junction (GEJ) and the herniation of the fundus through the hiatus. Type III represents a combination of types I and II and makes up over 90% of paraesophageal hernias, while type IV involves the herniation of abdominal structures other than the stomach through the hiatus [4]. The manifestation of HH depends on the type and size. The most common manifestations are gastrointestinal, which include gastroesophageal reflux disease (GERD), which causes heartburn and regurgitation, noncardiac chest pain, and may lead to esophageal complications such as reflux esophagitis, strictures, Barrett's esophagus, and even esophageal cancer. Type II HH can lead to less common issues like gastric outlet obstruction, gastric volvulus, and intestinal problems, and types III and IV are associated with cardiac and pulmonary presentations. As hiatal hernias enlarge and move further into the thorax, non-gastrointestinal symptoms such as dyspnea, atrial fibrillation, and even pulmonary fibrosis may become more frequent [5]. We are reporting a case of an elderly patient with a known history of hiatal hernia presenting with episodes of recurrent syncope.

## Case presentation

A 92-year-old female patient with a significant medical history of a medium-sized HH arrived at the hospital due to recurring episodes of syncope, which she had been experiencing for the past four weeks. These episodes were consistently triggered by heavy meals. The patient denied any prodromal symptoms, bowel or bladder incontinence, tongue biting, or seizure-like activity during these episodes. On examination, the patient was hemodynamically stable, orthostatic vital signs were negative, and the physical exam was unremarkable. 

**Figure 1:**
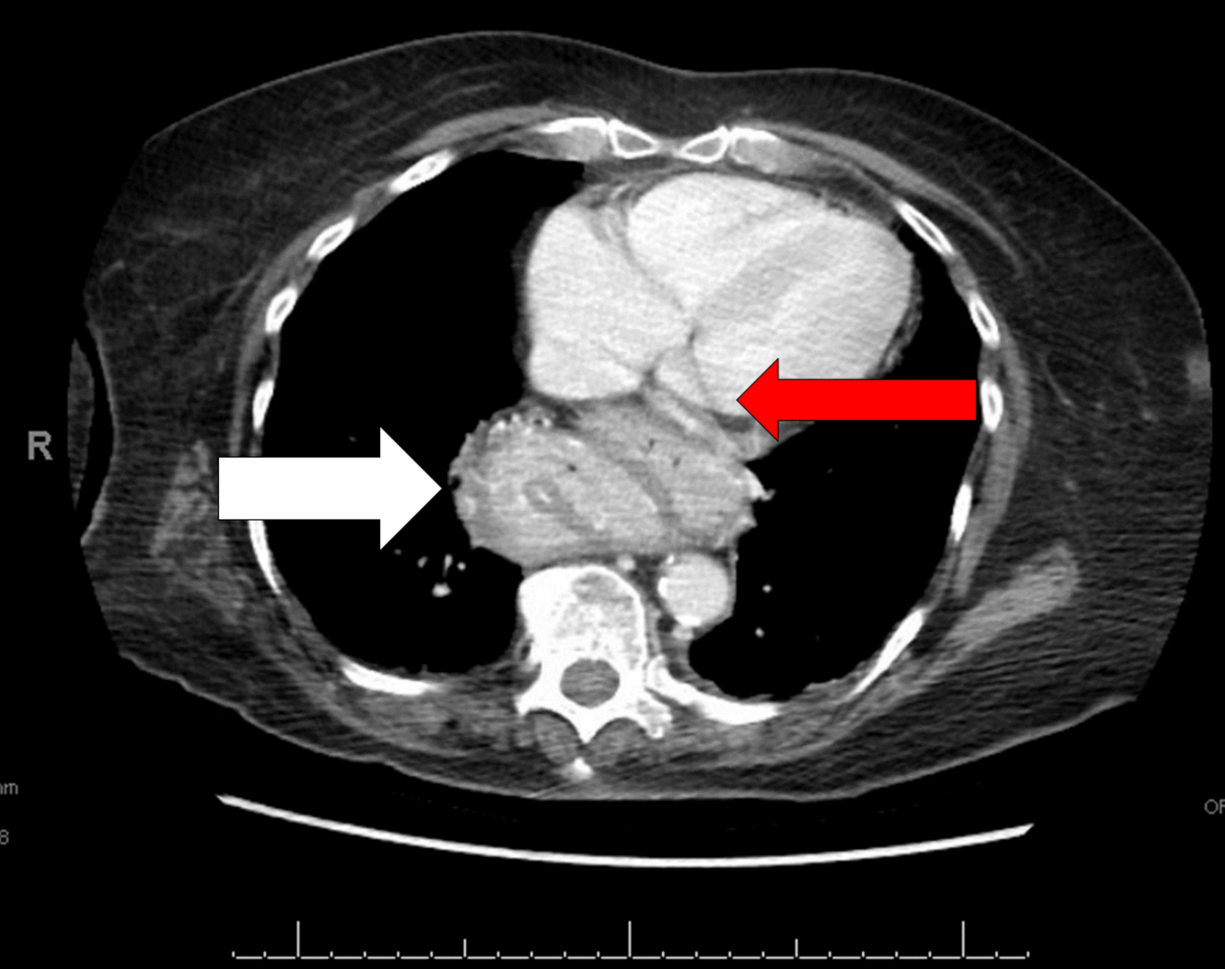
Large hiatal hernia with intrathoracic portion adjacent to left atrium

**Figure 2:**
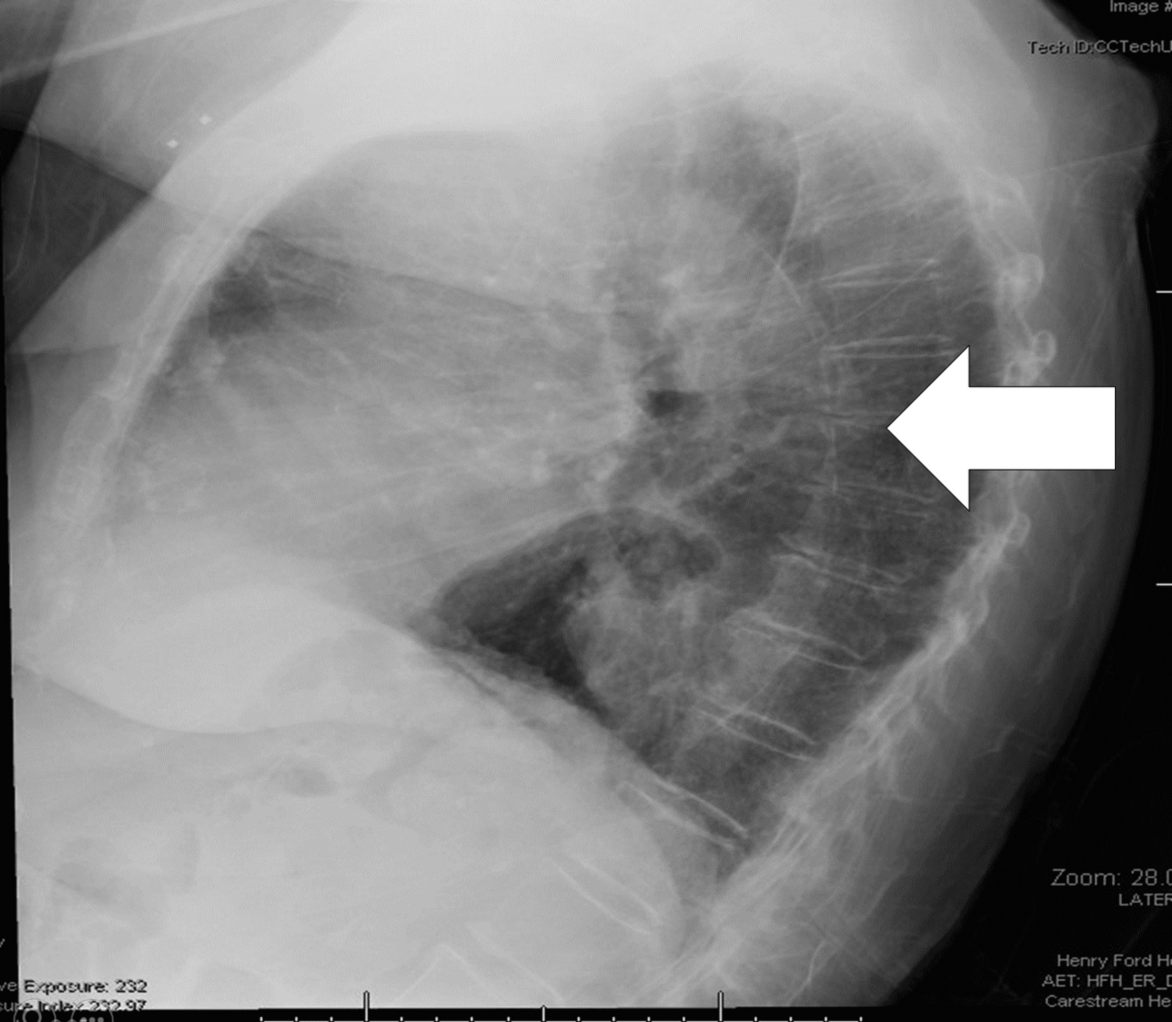
Lateral radiograph of moderately sized hiatal hernia from three months prior to presentation

Cardiac examinations, including EKG, echocardiography, and cardiac telemetry, were conducted to investigate potential heart-related causes. The results revealed that the patient's EKG and telemetry were within the normal range. However, the echocardiogram detected a minor grade I left ventricular diastolic dysfunction with an ejection fraction (EF) of 60%, deemed insignificant in the broader context of the individual's medical condition. Laboratory work revealed the presence of baseline anemia (hemoglobin level of eight). A CT scan of the brain was ordered to assess for any neurological abnormalities that could be contributing to the patient's symptoms, but no significant findings were observed. CT scan of the chest, abdomen, and pelvis (Figure 1) showed a large HH with a largely intrathoracic stomach adjacent to the left atria (LA) (white arrow) without gastric outlet obstruction. The HH was found to be compressing the left atrium (red arrow). Her hiatal hernia was first reported on a chest X-ray three months prior to her symptomatic presentation (Figure 2). 

After a thorough evaluation, the patient was not deemed a suitable candidate for surgical intervention and was provided with comprehensive education about her condition. She was discharged from the hospital with a prescription for proton pump inhibitors and was advised to consume small, frequent meals. Following her discharge, the patient continued to follow up with the same provider, and there were no reports of recurrent syncope episodes.

## Discussion

Esophageal hiatal hernia is marked by abnormal abdominal entry into the thoracic cavity. It is characterized according to the orientation of the esophageal junction and the diaphragm. The most common kind is a sliding hiatal hernia (type I), which arises from the right crus of the diaphragm. Age and a higher BMI are important risk factors, and congenital skeletal abnormalities exacerbate the risk via intestinal malrotations. Common symptoms include gastric reflux, nausea, bloating, chest and epigastric pain, pharyngeal and esophageal evacuation, and dysphagia. Melena and weight loss are serious signs [6]. 

Our patient is a 92-year-old female who arrived at the hospital with a complaint of syncope for the past four weeks, and her CT scan revealed a large hiatal hernia. Large hiatal hernias can result in significant posterior cardiac compression and atrial arrhythmias have also been described due to impingement on the left atrium [7,8]. The left atrium's anatomical proximity to herniated stomach contents increases the likelihood of mechanical irritation of the atria, autonomic neuronal connections, or inflammation, which may increase the risk of atrial fibrillation (AF) [9]. Symptomatic left atrial compression is a rare clinical condition that manifests as heart failure, syncope, or shock. A Medline search for "left atrial compression" yielded just 271 results. Only 17 cases of left atrial compression caused by gastrointestinal structures were discovered, 11 of which had hiatal hernia [10]. Hiatal hernias are frequently associated with gastroesophageal reflux disease (GERD) and esophagitis; Huang et al. reported an increased risk of atrial fibrillation in patients with GERD as an independent predictor [11,12]. Saito et al. were the first to report a case of a large hiatal hernia that compressed the heart and resulted in dynamic circulatory changes and syncope [13]. Various conditions can induce syncope episodes; beverages such as hot or cold carbonated drinks and some foods are all known factors, but left atrial compression is the most significant cause [1].

Conclusively, left atrial compression by the herniated abdominal contents can lead to syncope, which is diagnosed by a CT scan and successfully managed by PPIs or antihistamines (H2 blockers). However, if symptoms persist or if the patient develops obstructive symptoms, then surgical management with Nissen fundoplication may be warranted [14]. Our patient showed no signs of syncope on the subsequent follow-up, proving good adherence to the lifestyle modifications and medications (PPIs).

Further research and case studies may help elucidate the relationship between hiatal hernias and syncope, contributing to improved diagnostic and therapeutic strategies for patients with similar presentations.

## Conclusions

This case highlights the importance of considering structural anomalies such as hiatal hernias in the differential diagnosis of syncope, especially in cases where other more common causes have been ruled out. While hiatal hernias are typically associated with gastrointestinal symptoms, they can lead to unusual cardiac manifestations, including atrial arrhythmias, due to mechanical irritation of the left atrium.

The successful management of this patient involved a comprehensive evaluation, ruling out other potential causes, and a non-surgical approach with proton pump inhibitors and dietary modifications. This case report underscores the significance of individualized patient care and the need for healthcare providers to remain vigilant for atypical presentations of common conditions, especially in elderly patients.
